# Takotsubo syndrome – adding pieces to a complex puzzle

**DOI:** 10.1186/s12872-017-0731-y

**Published:** 2017-12-20

**Authors:** Athanassios Manginas, Angelos G. Rigopoulos, Boris Bigalke, Stefanos Sakellaropoulos, Muhammad Ali, Sophie Mavrogeni, Michel Noutsias

**Affiliations:** 1 Interventional Cardiology and Cardiology Department, Mediterraneo Hospital, Ilias Street 8-12, GR-16675 Glyfada, Greece; 2 Department of Internal Medicine III, Division of Cardiology, Angiology and Intensive Medical Care, University Hospital Halle, Martin-Luther-University Halle, Ernst-Grube-Straße 40, 06120 Halle (Saale), Germany; 3 Department of Cardiology, Charité - Universitätsmedizin Berlin, Campus Benjamin Franklin (CBF), Berlin, Germany; 4 Department of Cardiology, Asklepios Harzklinik Goslar, Goslar, Germany; 5 Department of Cardiology, Onassis Cardiac Syrgery Center, Leoforou Andrea Sygrou 356, Kallithea, 17674 Athens, Greece

**Keywords:** Cardiac magnetic resonance imaging, Clinical presentation, Heart failure, Takotsubo syndrome, Treatment

## Abstract

Takotsubo syndrome, a form of acutely decompensated heart failure, has drawn interest because of its intriguing pathophysiology and therapeutic dilemmas. In their recent work in BMC Cardiovascular Disorders, Abanador-Kamper et al. describe the therapy management in these patients and add valuable information on cardiovascular magnetic resonance imaging evolution.

## Letter to the Editor:

We read with great interest the publication by Abanador-Kamper et al. [1], in which the investigators report on the clinical outcome and the medical therapy used in every day practice. In this investigation on 72 patients with Takotsubo syndrome (TTS), the medical treatment followed the recommendations for acute coronary syndrome and acute decompensated heart failure (ADHF). The reported in-hospital complications rate was 5.6%, and the 2-year major adverse cardiac events (MACE) was 12%. In-hospital mortality was only 1%, and the mortality at 2 and 3 years were 5 and 8%, respectively. The authors attribute this favorable outcome, at least partly, to a more intense use of medications right with the onset of the TTS. It should be noted though that, in the present small and retrospective study, long term double and single antiplatelet therapy yielded comparable MACE rates of 6 and 4%, respectively. In contrast, in the much larger International Takotsubo Syndrome Registry of 1750 patients, in-hospital complications were observed in 21.8% of patients, while the rate of major adverse cardiac and cardiovascular events during the first 30 days after admission was 7.1%, with a mortality of 5.9%. During follow-up, mortality was 5.6% per patient-year [2]. TTS was previously believed to have a relatively benign prognosis, however, is now regarded as a form of acute, reversible, heart failure syndrome with potentially serious clinical complications. A recent Position Statement from the ESC Heart Failure Association aids in clinical decision-making and describes the unmet needs for evidence-based therapeutic strategies [3]. Useful data on prescription trends provided by the present study is for example the wide use of long-term antiplatelet treatment for a syndrome more closely resembling ADHF.

The authors should also be congratulated for providing cardiovascular magnetic resonance imaging (CMR) in the acute phase (2 days) and at follow-up (2.3 months) in all TTS patients. CMR is an excellent diagnostic modality and, if available, may help in the differential diagnosis between previous myocardial scar and TTS (LGE present or absent respectively) and between acute myocarditis and TTS. In addition, it is more accurate in the detection of apical thrombus, and for the diagnosis of right ventricular involvement. It is regarded complimentary to echocardiogram for documentation of myocardial recovery.

Although first reported in 1990, TTS offers many areas for future investigation, already touched upon by clinicians and scientists. How can the different anatomical variants of TTS be explained [4]? Is the brain (hypothalamic-pituitary-adrenal)-heart interaction predisposing patients to TTS [5]? What is the significance of blood hyperviscocity [6], autonomic imbalance [7] or endothelial dysfunction [8] and could they become therapeutic targets? Besides conservative medical therapy, could psychological or behavioral counseling prevent TTS or its recurrences? Finally, are there any longterm consequences and/or risk from recurrences?

Future randomized studies, ideally through national or international TTS collaborations, will certainly advance the field in this fascinating syndrome.

## Abbreviations

ADHF: Acute decompensated heart failure
CMR: Cardiac magnetic resonance
LGE: Late gadolinium enhancement
MACE: Major adverse cardiac events
TTS: Takotsubo syndrome
